# P095 Acquired Bartter syndrome secondary to subclinical Sjögren’s disease with a positive anti-Ro antibody, unmasked during pregnancy: a case report

**DOI:** 10.1093/rap/rkag113.125

**Published:** 2026-11-03

**Authors:** Joseph Shaw

**Affiliations:** Rheumatology, Great Western Hospital, Swindon, United Kingdom

## Abstract

**Introduction:**

Bartter syndrome is a rare, hereditary tubulopathy characterised by impaired sodium and chloride reabsorption in the loop of Henle, leading to hypokalaemia, metabolic alkalosis, hyperreninaemia and hyperaldosteronism ^(1)^. Acquired Bartter syndrome results in the phenotype of hereditary Bartter syndrome, without a predisposing genetic mutation ^(2,3)^. Primary Sjögren’s Disease (SD) is a systemic autoimmune condition characterised by lymphocytic infiltration of exocrine glands. In rare cases, acquired Bartter syndrome has been documented as an extra-glandular complication of SD ^(4,5,6,7,8)^. This case report details a young woman with an asymptomatic positive anti-Ro antibody, presenting with acquired Bartter-like syndrome, unmasked by pregnancy.

**Case description:**

A 39-year-old woman at 14 weeks gestation was referred to antenatal endocrinology clinic in December 2024, with persistent hypokalaemia since September 2023 (2.75–3.4 mmol/L). She denied vomiting or diarrhoea and exercised regularly, had a family history of Graves’ disease and took regular oral iron replacements. She had a previous miscarriage at 16 weeks and a caesarean section in February 2021 following diagnosis of gestational diabetes with no documented electrolyte disturbance during this pregnancy.

Blood tests again demonstrated hypokalaemia (3.23 mmol/L), hypomagnesaemia (0.65 mmol/L), hyponatraemia (134 mmol/L) and random urinary potassium of 94 mmol/L. On examination, there were no clinical signs of Cushing’s syndrome or thyrotoxicosis. Oral potassium replacements were prescribed.

In February 2025, severe hypokalaemia (2.13 mmol/L) led to hospital admission for intravenous potassium replacement. Regular oral potassium was increased with oral magnesium supplements commenced. A positive anti-Ro/SSA antibody titre was identified in March 2025, prompting referral to Rheumatology clinic. The patient gave birth in May 2025 with a normal foetal cardiac screen, no evidence of foetal heart block or neonatal lupus. Hypokalaemia, hyponatraemia and hypomagnesaemia improved post-partum on regular oral replacement.

In June 2025 a venous blood gas demonstrated hypokalaemic, hypochloraemic metabolic alkalosis (pH 7.507, bicarbonate 41.3mmol/L). Rheumatology review in July 2025 found no sicca symptoms, skin rashes, synovitis, or clinical features of a systemic autoimmune connective tissue disease on examination.

Nephrology review in October 2025 included genetic testing for inherited tubulopathies which was negative. A 24-hour urine collection demonstrated marked kaliuresis (272 mmol/24 hours) with urinary calcium within normal limits (2.6 mmol/24 hours). As of June 2026, the patient remains stable on oral potassium replacement and magnesium supplementation, with most recent serum potassium 4.1 mmol/L and magnesium 0.74mmol/L.

**Discussion:**

Persistent hypokalaemia, marked kaliuresis, hypomagnesaemia, hypochloraemia and hyponatraemia, along with metabolic alkalosis, are characteristic of a renal salt-wasting tubulopathy ^(9)^. The negative genetic testing for inherited tubulopathies refutes the diagnosis of hereditary Gitelman’s or Bartter syndrome, in keeping with an acquired mechanism. The hyponatraemia, normocalciuria and mild hypomagnesaemia favour an acquired Bartter syndrome.

In SD-related acquired Gitelman’s and Bartter syndrome, an underlying mechanism is yet to be established. However, the pathophysiology of SD-related distal renal tubular acidosis (dRTA) involves lymphocytic infiltration and subsequent tubulointerstitial nephritis (TIN), which disrupts collecting-duct architecture and impairs hydrogen ion secretion ^(10)^. By extrapolation, similar lymphocytic infiltration and TIN involving the loop of Henle could interfere with NKCC2 cotransporters, resulting in an acquired Bartter-like phenotype. To further support this, a renal biopsy would be required. However, as the patient is currently clinically stable, the potential benefits do not outweigh the procedural risks.

A nuance of this case was the asymptomatic nature of the anti-Ro antibody. Detection of an anti-Ro antibody without overt sicca symptoms is classified as subclinical SD and may precede symptoms by many years ^(11)^. TIN can also occur in subclinical SD, before the onset of sicca symptoms ^(12)^. This patient presented with features of an acquired renal tubulopathy, and while under investigation, was shown to have anti-Ro antibodies, hence being the presenting feature of this patient’s SD.

Another unique feature of this case was the overlap with pregnancy. The physiological changes that occur during pregnancy can exacerbate existing renal tubulopathies due to upregulation of the renin-angiotensin-aldosterone system (RAAS), plasma volume expansion and increased glomerular filtration rates ^(13)^. Hypokalaemia is a known risk factor for poor foetal outcomes, including premature delivery and foetal demise ^(14)^. This case highlights the importance of investigating for underlying autoimmune tubulopathies in unexplained hypokalaemia, particularly during pregnancy.

**Key learning points:**

• This case highlights several important clinical lessons in the assessment and management of electrolyte disturbances, particularly in pregnancy. Persistent hypokalaemia should not be solely managed with oral replacement without thorough prior investigation. Instead, it warrants a structured diagnostic approach that includes urinary electrolyte analysis, acid–base evaluation and consideration of renal tubular causes. Failure to investigate appropriately risks overlooking significant underlying pathology ^(16)^.

• A second key learning point is the recognition that acquired Bartter syndrome can occur as an extra-glandular manifestation of primary SD ^(4,5,6,7,8)^. Although classically associated with sicca symptoms, SD can initially present with renal involvement, possibly including salt-wasting tubulopathies as in this case ^(15)^. In subclinical SD, this renal phenotype may precede the development of glandular symptoms by months or even years, underscoring the importance of autoimmune screening in cases of persistent hypokalaemia.

• In the case of undifferentiated hypokalaemia, pregnancy adds further complexity, as physiological adaptations such as RAAS upregulation, plasma-volume expansion, and increased glomerular filtration can unmask or exacerbate underlying tubulopathies ^(13)^. These changes can exacerbate renal potassium and magnesium losses, placing both mother and foetus at risk, as demonstrated in this case with significant peripartum electrolyte derangements (Potassium nadir 2.13mmol/L). Given the association between maternal hypokalaemia and adverse foetal outcomes, close monitoring and proactive correction of electrolyte abnormalities are essential throughout pregnancy, especially in cases of renal tubulopathies ^(14)^.

• In conclusion, we present a case of acquired renal tubulopathy as the primary presenting feature of SD. Anti-Ro antibody production and extra-glandular manifestations of SD can precede sicca symptoms by many years and clinicians should be aware of biochemical findings suggestive of a renal tubulopathy. Early recognition of autoimmune-mediated renal disease allows timely multidisciplinary management and may prevent long-term complications ^(10)^.

